# The electrical conductivity of CNT/graphene composites: a new method for accelerating transmission function calculations

**DOI:** 10.3762/bjnano.9.117

**Published:** 2018-04-20

**Authors:** Olga E Glukhova, Dmitriy S Shmygin

**Affiliations:** 1 Saratov State University, Saratov, Russian Federation

**Keywords:** carbon composites, electronic properties, interpolation, quantum transport, transmission function

## Abstract

We present a new universal method to accelerate calculations of transmission function and electrical conductance of 2D materials, the supercell of which may contain hundreds or thousands of atoms. The verification of the proposed method is carried out by exemplarily calculating the electrical characteristics of graphene and graphane films. For the first time, we calculated the transmission function and electrical conductance of pillared graphene, composite film of carbon nanotubes (CNTs)/graphene. The electrical conductance of different models of this material was calculated in two mutually perpendicular directions. Regularities in resistance values were found.

## Introduction

The development of technologies for the synthesis of graphene nanomaterials has led to an expansion of the scope of their application. One of the graphene composites that have been actively studied in the last few years is pillared graphene [1]. It is a graphene layer, connected seamlessly by single-walled carbon nanotubes (SWCNTs). One of the advantages of this material is its high strength and resistance to mechanical stress [2–4]. In combination with high electrical capacity and efficient electronic transfer between graphene sheets, this nanomaterial has already been recognized as promising as an electrode for storage batteries and supercapacitors [5–7]. There remain many questions about the conductive properties of pillared graphene and their dependence on the length and diameter of the nanotubes. At the moment, there is no experimental data on the conductivity of pillared graphene, so the theoretical prediction of the transmission regularities in this material is relevant. However, the calculation of the electrical conductance of pillared graphene by quantum mechanical methods is difficult due to its large supercell. These calculations require too much time, even when using modern computing tools.

The non-equilibrium Green function (NEGF) method with density functional tight-binding (DFTB) scheme or density functional theory (DFT) scheme is used to calculate the electrical conductance of molecular structures consisting of atoms of various elements with high accuracy [8]. Within the NEGF formalism, each system represents left and right electrodes and the molecules between them. The probability that an electron will transmit from the left to the right electrode is described by the transmission function *T*(*E*). The dependence of the transmission function on the energy of the electron in the system is characteristic for each point of the reciprocal lattice. The final form of *T*(*E*) depends on the number of *k*-points in the reciprocal lattice, because *T*(*E*) average over these lattice points. Using the convergent transmission function it is possible to find the electrical conductance of the nanostructure. However, in order to obtain a converged form of the averaged transmission function, it may be necessary to calculate it in a set of points in the reciprocal lattice, which is unattainable for a large number of atoms in the considered system. In this connection, the development of methods for accelerating the calculation of the transmission function without a significant loss in the accuracy of calculations has particular relevance and significance for research of the electrical conductive properties of new composite materials. At present, such accelerating techniques are practically absent. We found only one work [9] in which the authors attempted to propose an algorithm that accelerates the calculations of the transmission function of large systems. However, the solution proposed in above mentioned paper did not significantly increase the computational speed, which is especially critical at considering new carbon composite materials such as pillared graphene and other varieties of graphene–nanotube structures. The purpose of this work is to propose an alternative approach to the calculation of transmission function and electrical conductance of composite nanomaterials, which allows us to investigate the electrophysical properties of atomic structures with hundreds and thousands of atoms in the supercell. The verification of the proposed approach is carried out by the example of calculations of the transmission function and electrical conductance of perspective 2D carbon materials, namely graphene, graphane and a graphene–carbon nanotube hybrid composite.

## Computational Details

In order to calculate the electrical conductance we use the Green–Keldysh functions and the Landauer–Büttiker formalism [8]. The calculation of energy and band structure is carried out by the DFT method in the tight-binding approximation [10–12] within the Kvazar software package [13]; the parametrization pbc-0-3 was used [14–16]. The electrical conductance is described by the expression:

[1]
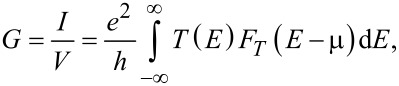


where *T*(*E*) is the transmission function characterizing the quantum mechanical transparency of the conducting channel as a function of the energy of the electron moving along it, μ is the Fermi energy of the electrode, *e* is the charge of the electron, and *h* is the Planck constant. The thermal broadening function *F_T_*(*E*) is calculated by the formula:

[2]
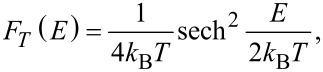


where *k*_B_ is the Boltzmann constant, and *T* is the temperature. The transmission function is given by:

[3]
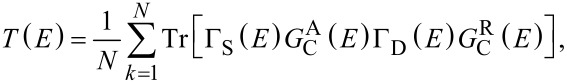


where 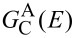 and 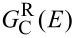 are, respectively, the advanced and retarded Green matrices (describing the contact with the electrodes), Γ_S_(*E*) and Γ_D_(*E*) are, respectively, the level broadening matrices for source and drain. The function *T*(*E*) is found by summation over the entire Brillouin zone (BZ) and *N* is the number of points in the reciprocal space. The accuracy of calculating *T*(*E*) is determined by the segmentation of the reciprocal space and the considered number of energies values. The calculation will be reliable when the function *T*(*E*) does not change with decreasing pitch of the change in the wave number *k*. For example, for finitely segmented BZ with limits *a* and *b*, the function *T*(*E*) converges to its true form for *N* ≥ 10^3^. For materials in which the cell contains of the order of several hundreds or thousands of atoms, the calculation of *T*(*E*) at a single point (for a fixed *k*) takes a rather long time, thus making 10^3^ calculations impossible. For example, for a 2D crystal cell with 472 atoms, the calculation of the transmission function averaged over 288 points of the reciprocal space takes almost four days for the parallel calculation in 24 processes (Intel^®^ Xeon^®^ CPU E5-2660 v2 with a frequency of 2.2 GHz). If the BZ is not a segment, but a 2D or 3D figure, the number *N* increases by orders of magnitude. In the next section, a method will be described to reduce the number of reciprocal space points and energy values for which a transmission function calculation is required, without substantially losing accuracy of the shape of *T*(*E*).

## Results and Discussion

### Description and verification of the method for accelerating transmission-function calculations

**Figure 1:**
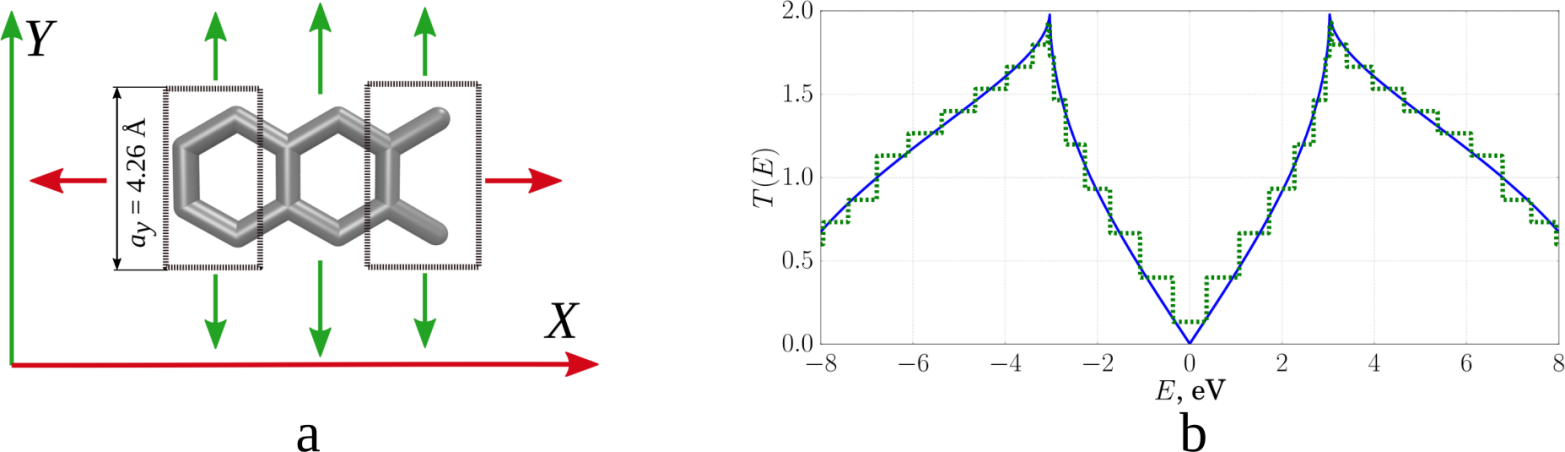
Graphene: a) unit cell with electrodes; b) transmission function (green: d*k* = 0.1 1/Å, blue: d*k* = 0.0015 1/Å).

Let us demonstrate the proposed method for calculating *T*(*E*) for two different *N* by using the example of a graphene monolayer. Figure la shows the considered system, that is, the unit cell connected to the electrodes. Since we calculated the conductance of this material (graphene), the same cells act as electrodes. Electrodes are translated to infinity along the *Y*-direction, and they are semi-infinite along *X*. The central cell is also translated to infinity over *Y*. Thus, the wave number *k_y_* varies within (−π/*a_y_*; π/*a_y_*), where *a_y_* is the unit cell size along the *Y*-direction (as shown in Figure 1a, the software VMD [17] was used for visualization). The calculated functions *T*(*E*) from Equations 1–3 with different values of *N* are shown in Figure 1b. For a small step of the decomposition d*k* = 0.1 1/Å (*N* = 15), the function has a step-like form. When the step of the decomposition is reduced to d*k* = 0.0015 1/Å (*N* = 984), the curve takes the correct well-known form for a graphene monolayer. The transmission function is represented in conductance quanta *e^2^*/*h*.

For each point of the reciprocal lattice, the calculated *T*(*E*) function is additionally processed. The values of *T*(*E*) near the transition area between the steps are refined to make them more abrupt. In many cases, this procedure allows us to eliminate the need for a more detailed decomposition of the energy interval. For this, within transmission-function calculations we used an interval-halving technique, which allows one to obtain a 16-times more accurate boundary value between the steps for four iterations. Each iteration reduces the difference between the energies *E*_1_ and *E*_2_ by a factor of two (*E*_1_ is the last energy value for a certain *k*-point at which *T* = *T*(*E*_1_), *E*_2_ is the first energy value for the same *k*-point at which *T* = *T*(*E*_2_) and *T*(*E*_2_) ≠ *T*(*E*_1_)), since the value of the transmission function at the midpoint *E*_3_ = 0.5(*E*_1_ + *E*_2_), which is either *T*(*E*_1_) or *T*(*E*_2_), is determined. Then, the interval (*E*_1_, *E*_3_) is considered if *T*(*E*_3_) = *T*(*E*_2_), or the interval (*E*_3_, *E*_2_) if *T*(*E*_3_) = *T*(*E*_1_). Therefore, four similar iterations yield a 16-fold reduction of the energy difference between two adjacent steps of the transmission function.

**Figure 2:**
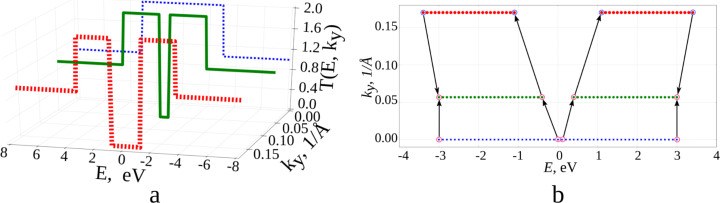
Transmission function: a) *Т*(*Е*) for three different *k_y_*; b) the distribution of points of the transmission function for different energies and quantities *k_y_* for a fixed value of *Т*(*Е*) = 2.

To eliminate the step-like form of the averaged transmission function, we interpolate the function *T*(*E*) between two neighbouring points of the reciprocal lattice. Figure 2a shows step plots of *T*(*E*) for three different numbers *k_y_*. In general, independently of *k_y_*, interpolation for each point of the polygonal chain determines the nearest points with energy having the same value of the function *T*(*E*) and belonging to the neighbouring polyline. The picture of the distribution of points in this case has the form shown in Figure 2b, when one value of *T*(*E*) = 2 is fixed. Thus, all the points of neighbouring polygonal lines having the same value of *T*(*E*) are first found, and then the nearest ones are selected from the energy difference. If a polyline has two adjacent lines, then each point of this polyline can have no more than one near point on each of them. Similarly, in the case of a single neighbouring polyline, there will not be more than one near point for each point on the polygonal chain. If the difference in the values of the transmission function exceeds one, additional points are added. For example, if for some value of *k_y_* the transmission function undergoes a jump on passing from a point with energy *E_i_* to a point with energy *E_i_*_+1_, with *T_i_*_+1_ − *T_i_* = 2, then an additional point will be added to this polygonal chain. This point is characterized by an energy value of 0.5 (*E_i_*_+1_ + *E_i_*) and the transmission function at this point is equal to *T_i_* +1. This is necessary in order to find the nearest point with the value of the transmission function *T_i_* + 1.

Next, the nearest points found are used to add additional points between them, lying on the segment connecting these points. All additional points have the same value of *T(E)* as the nearest points. The number of points added depends on the length of the segment connecting the nearest points on different polygonal chains. The longer the lengths of the segment, the more additional points are added. If an additional point has not been used to form the connecting segment, it is not used to construct an interpolating function. The starting points and the remaining added points are used to construct the interpolating function.

**Figure 3:**
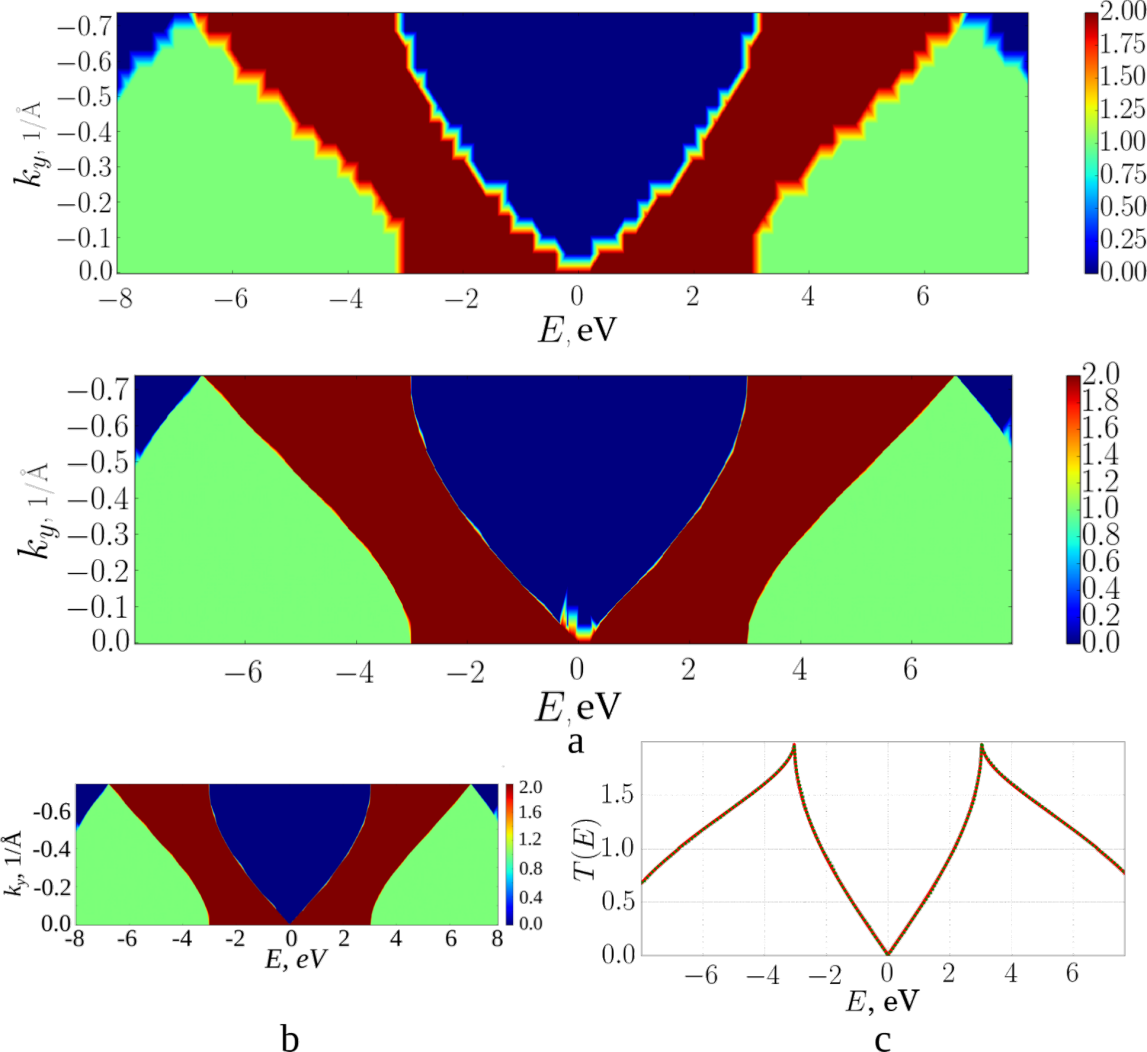
Graphene transmission function: a) a map of *T*(*E*) for the initial partitioning over *k_y_* (from above) and after applying the interpolating function *Т*_1_(*Е*, *k_y_*); b) a map of *T*(*E*) after applying the interpolating function *Т*_2_(*Е*, *k_y_*); c) averaged *T*(*E*) over all *k_y_* (green: interpolated, red: converged).

The interpolating two-dimensional function *T*_1_(*E*, *ky*) makes it possible to realize a detailed decomposition over *k_y_* values, ensuring smoothing of the initial roughness. Figure 3a shows maps of the transmission function *T*(*E*): Figure 3a, top – before the interpolation procedure (there are additional points obtained by interval-halving technique); Figure 3a, bottom – after applying the constructed interpolating function *T*_1_(*E*, *ky*). The step-like behaviour of the function *T*(*E*) has disappeared everywhere, except for the region near the Fermi level (0.0 eV here). Indeed, there may exist “special regions” for which the original partitioning by *k_y_* was too coarse. Special regions are determined by the researcher in the gradient of the image. Areas of smooth colour change indicate the lack of partitioning for this area. Calculations with a more particular partition are additionally carried out for this area, but already in a narrow range of values: from 0 to 0.2 1/Å for *k_y_*, and from −0.3 to 0.3 eV for *E*. The next step is the construction of a new interpolating function *T*_2_(*E*, *ky*). Figure 3b shows the map of the transmission function after applying *T*_2_(*E*, *ky*), Figure 3c show the function *T*(*E*) averaged over all *k_y_*. The solid curve shows the calculation with the distance between two neighbouring points in reciprocal lattice of 0.0015 1/Å, the dotted line shows the result of applying the developed method. The norm of the difference between the interpolated and the converged values is 0.68%. Time taken to obtain the average transmission function (with parallel calculation using eight processes on the Intel Xeon CPU E5-2690 v4 CPU with a frequency of 2.6 GHz) varies: 34 minutes, 2 seconds per accurate calculation and 2 minutes, 22 seconds to obtain the final values of the interpolated graph. Note that, in general, the time taken to add points depends on the amount of input data, and not on the number of atoms in the considered structure.

**Figure 4:**
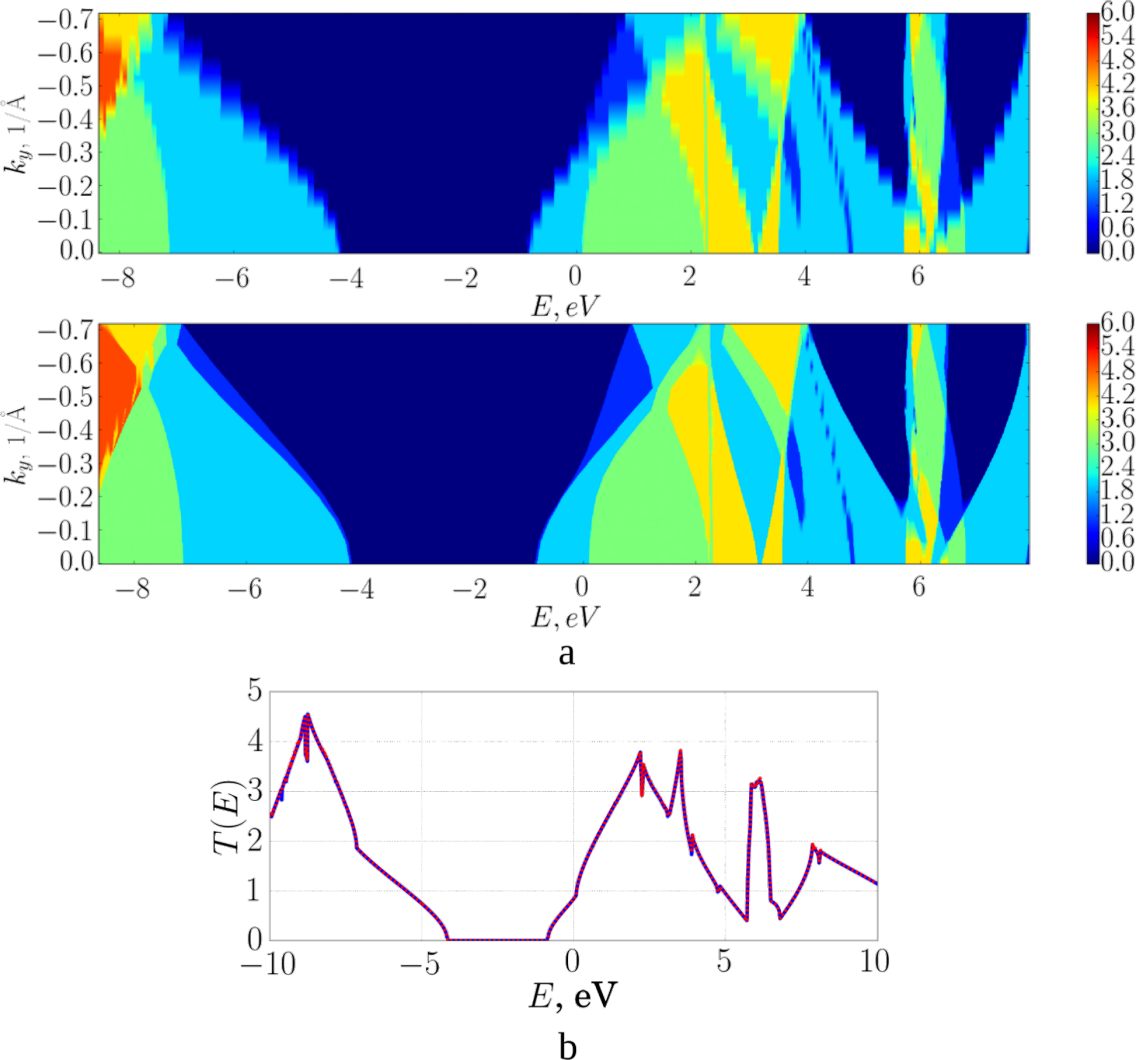
Graphane transmission function: a) a map of *T*(*E*) for the initial partitioning over *k_y_* (top) and (bottom) after applying the interpolating function *Т*(*Е*, *k_y_*); b) averaged *T*(*E*) over all *k_y_* (blue: interpolated, red: converged).

The proposed method for calculating the transmission function was also tested with the example of graphane. Figure 4 present the results of the study of a graphane fragment using the proposed method. The number of points for accurate calculation is 720, for the rough approximation it is 24. The calculation times for the transmission function (for parallel calculation using eight processes on the Intel Xeon E5-2690 v4 CPU with a frequency of 2.6 GHz) for accurate calculation and interpolation are 146 minutes, 29 seconds and 7 minutes, 21 seconds, respectively. The norm of the difference between the interpolated values and the ones obtained from the direct calculation is 1.79%.

The results of solving the test problems show that the transmission function calculations were accelerated by factor of 14.38 for graphene and of 19.92 for graphane by using the method developed here. Nevertheless, the speed of calculations is limited by the complexity of the dependence of the transmission function on energy and the considered point of the reciprocal lattice. The higher the rate of change in the transmission function, the more detailed calculations are needed.

The accuracy of the results of the transmission function calculations obtained using the proposed method depends on the size of the unit cell and the chosen reciprocal lattice vector *k*. The test problems solved for graphene and graphane show that the discrepancy between the values of the transmission function calculated without the developed method and with its application was about 1–2%.

### Transmission function and conductance of 2D graphene/CNT composites

**Figure 5:**
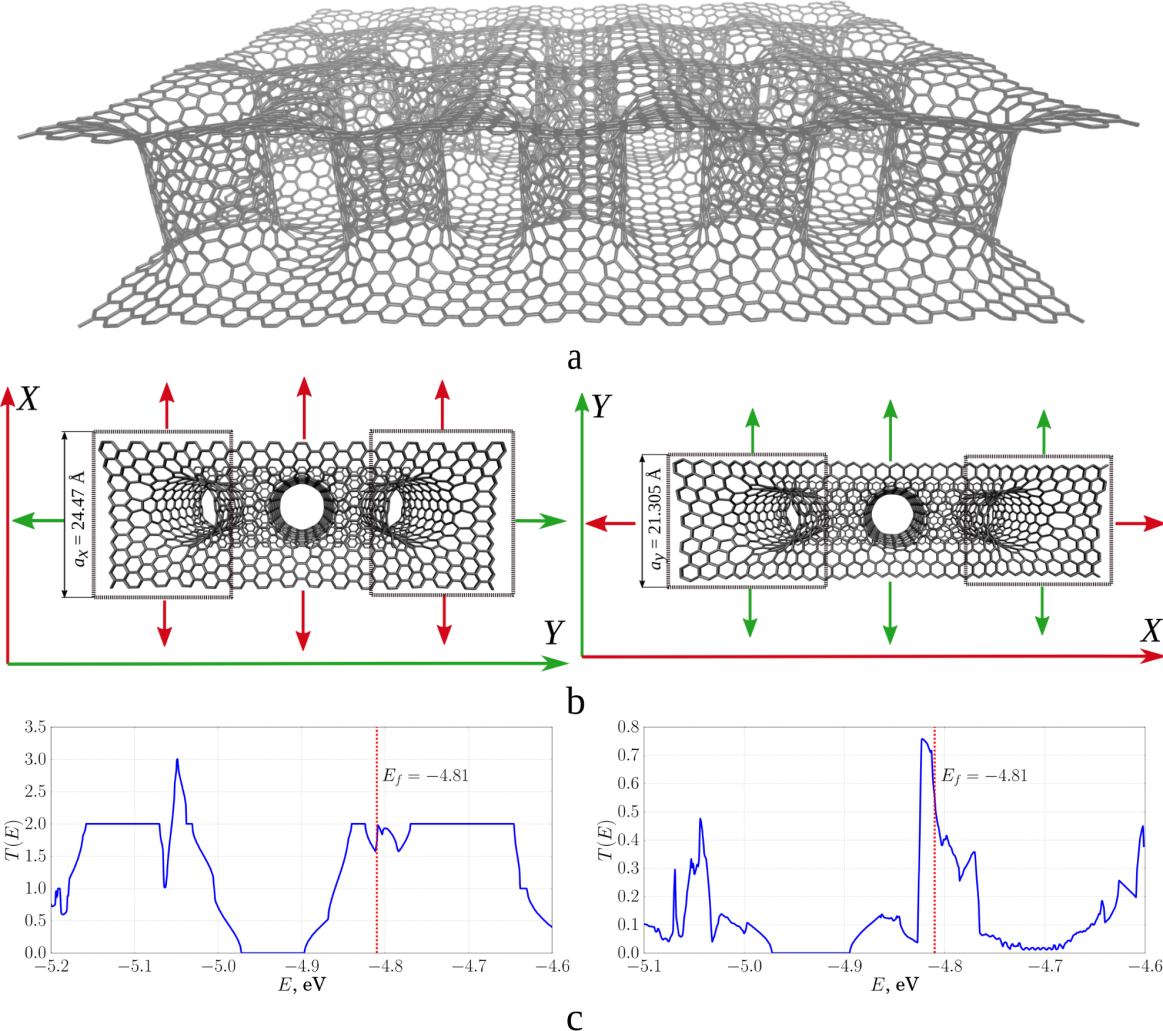
2D composite of pillared graphene based on CNT (9,9) with a length of 2.4 nm: a) atomic structure; b) schematic representation of the cell of the composite and electrodes in the case of electron transport along *X* (left) and *Y* (right); c) transmission functions along the *X*- (left) and *Y*- (right) directions.

Using the developed method for calculating the transmission function we investigated the transmission functions and electrical conductance of 2D graphene/CNT composites. The investigated film was modelled by two layers of graphene connected by single-layer armchair tubes (9,9) with a diameter of 1.23 nm (tubes of diameter 1–1.5 nm are typical for such composite materials). The distance between the tubes was equal to 2.1 nm, the length of the tubes (i.e., the distance between the layers of graphene) ranged from 1.1 to 2.4 nm. The graphene sheet had a length of 2.45 nm along the *X*-axis and 2.13 nm along the *Y*-axis for each unit cell. Figure 5a shows the atomic structure of a pillared graphene film with an inter-tube distance of 2.1 nm. The tubes are connected seamlessly with graphene, i.e., the CNT smoothly passes into the graphene sheet, and the junction contains not only hexagons, but also defects in the form of pentagons, heptagons and octagons. In order to calculate the electrical conductance in the *X*- and *Y*-directions, as in the case of the graphene monolayer, a central supercell and supercell electrodes are separated (see Figure 5b). The conductance calculation scheme corresponds to the electronic transport along *X* (along the zigzag edge) in the left figure, and along *Y* (along the armchair edge) in the right figure. In this case the supercell consists of 580 atoms with a distance between the graphene layers of 2.4 nm. The conductance was calculated using the developed method because of the large number of atoms. The plots of the function *T*(*E*) averaged over all *k* in the case of electron transport in the *X*-and *Y*-directions are shown in Figure 5c.

Similarly, the transmission functions *T*(*E*) were calculated for all models of supercells of a composite on the basis of CNT (9,9) at the same distance between tubes of 2.1 nm. The conductance and resistance of the pillared graphene film were calculated based on the calculated *T*(*E*). Table 1 shows the corresponding data: tube length, number of atoms in the transmitted cell, calculated Fermi level, conductance and resistance. The Fermi level is in the interval (−4.88 eV; −4.73 eV), that is, it is shifted downward compared to ideal CNTs of the same diameter (−4.66 eV). Conductance and resistance behave non-monotonically. It can be said that the resistance oscillates around a value of 12 kΩ in the *Y*-direction for single-layer composites, and around a value of 90 kΩ in the *X*-direction only with larger amplitude.

**Table 1:** Data of investigated single-layer composite films and the results of modelling.

length of the tube, nm	number of atoms in the supercell of the composite	direction of translation	Fermi energy, eV	conductivity, μS	resistance, kΩ

0.60	400	*X*	−4.74	135.93	7.35
		*Y*		11.03	90.65
0.85	436	*X*	−4.88	32.48	30.79
		*Y*		8.32	120.12
1.10	472	*X*	−4.85	92.21	10.85
		*Y*		24.89	40.18
1.34	508	*X*	−4.73	142.96	6.99
		*Y*		10.41	96.05
1.59	544	*X*	−4.85	72.26	13.65
		*Y*		13.19	75.78
1.84	580	*X*	−4.81	122.08	8.19
		*Y*		19.8	50.5

**Figure 6:**
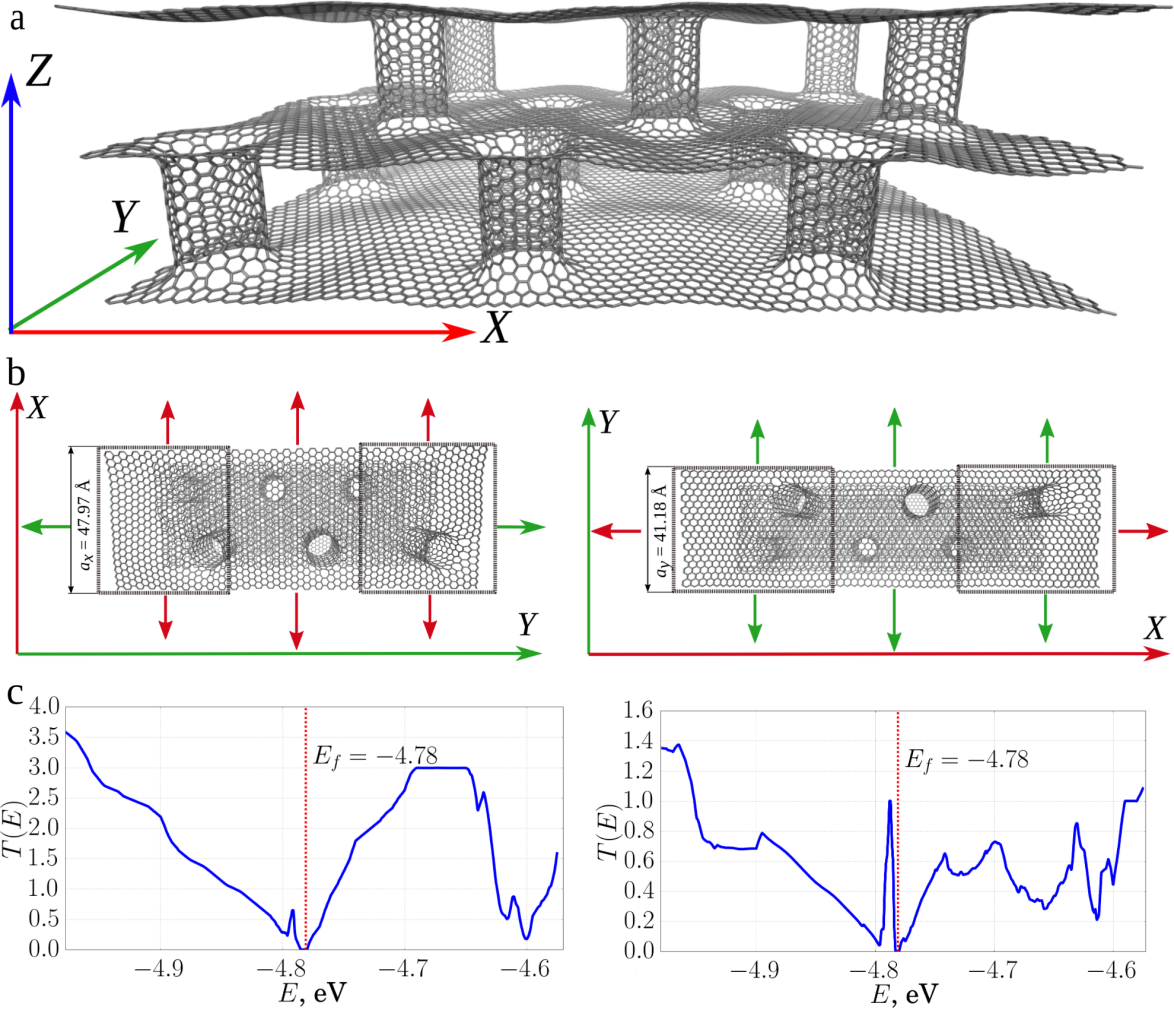
Two-layer 2D composite of pillared graphene based on CNT (9,9) with a length of 2.4 nm: a) atomic structure; b) schematic representation of the cell of the composite and electrodes in electronic transport along X (left) and along Y (right); c) transmission functions along the *X*- (left) and *Y*- (right) directions.

Similar investigations were carried out for two-layer pillared graphene (Figure 6). The graphene sheet length was 4.8 nm along the *X*-axis and 4.12 nm along the *Y*-axis for each unit cell.

The resistance for two-layer composites averages 10.2 kΩ in the *Y*-direction and 29.32 kΩ in the *X*-direction. The difference between the resistance in the *X*- and Y-directions is significantly lower for two-layer composite in comparison with single-layer composite. The conductivity in the *Y*-direction has increased for all the considered situations (see Table 2), while the conductance value decreased in the *X*-direction for a composite with nanotube lengths of 1.1 and 1.84 nm.

**Table 2:** Data of investigated two-layer composite films and the results of modeling.

length of the tube, nm	number of atoms in the supercell of the composite	direction of translation	Fermi energy, eV	conductance, μS	resistance, kΩ

0.60	2400	*X*	−4.712	141.26	7.08
		*Y*		41.66	24.00
1.10	2544	*X*	−4.798	84.82	11.79
		*Y*		30.30	33.00
1.84	2760	*X*	−4.781	86.06	11.62
		*Y*		32.31	30.95

**Figure 7:**
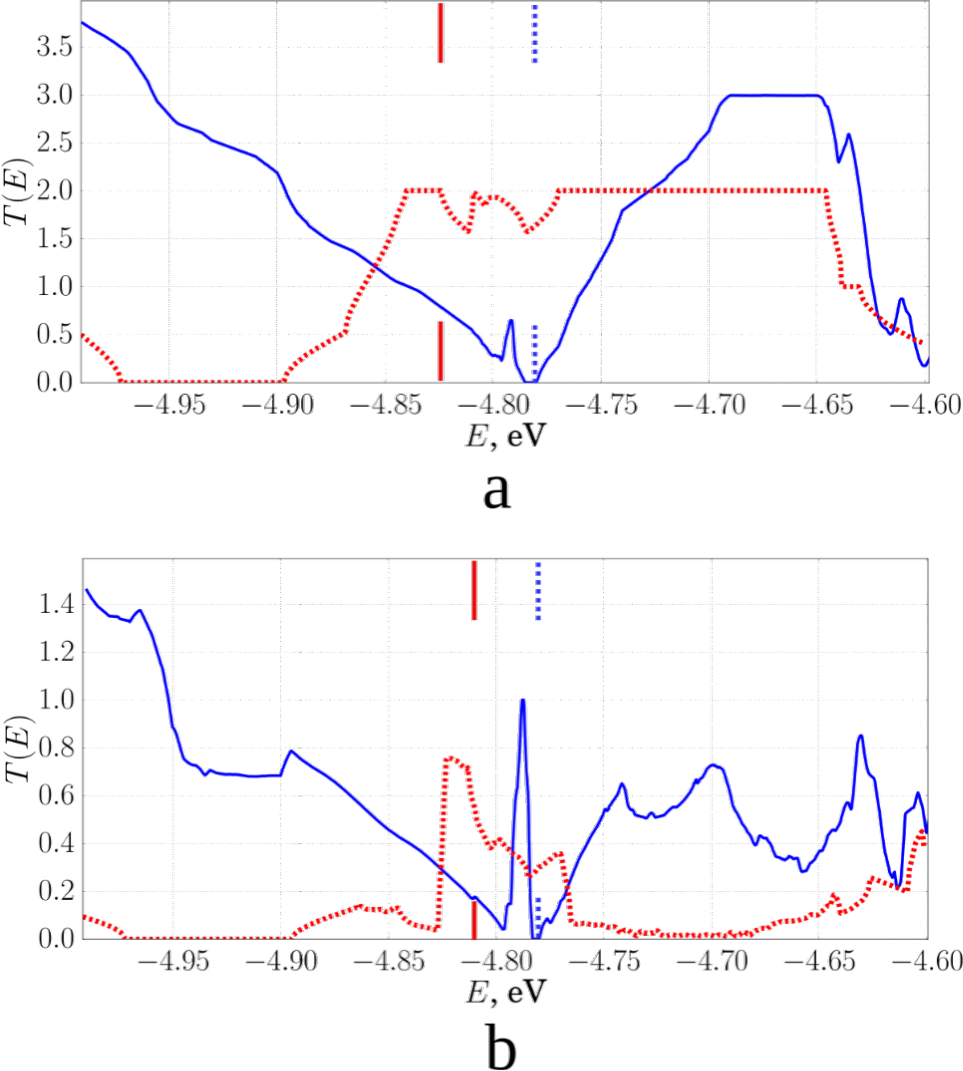
Plots of the transmission functions for two-layer (blue) and single-layer (red) composites (the tube length in the composite was equal to 1.84 nm): a) in the direction of the *X*-axis, along the zigzag edge; b) in the direction of the *Y*-axis, along the armchair edge. In addition, the values of the Fermi energy for composites are shown by short lines of the corresponding color.

A comparison of the plots of the transmission functions for single-layer and two-layer composite is shown in Figure 7.

Based on obtained results, we can conclude that for the single-layer pillared graphene film, the resistance in the direction of the *Y*-axis (zigzag edge of the graphene sheet) varies insignificantly and does not depend on the distance between the graphene layers. For a two-layer composite, the average resistance depends on the axis direction. This can be explained by the high electrical conductance of graphene nanostructures with a zigzag edge. The conductance of these ribbons does not depend on the width of the ribbon and weakly depends on its topology. In the direction of the *X*-axis (armchair edge of graphene sheet), the electrical conductance of single- and two-layer composites depends significantly on the topology of the film. The resistance changes drastically with the increase in the CNT length.

## Conclusion

We created a new universal method for calculating the electron-transmission function and electrical conductance at quantum transport in composite nanomaterials. This method allows us to investigate the electrophysical properties of atomic structures, which contain hundreds and thousands of atoms in the transmitted supercell. By the example of monolayer graphene and graphane it was shown that the developed method significantly reduces the calculation time of the transmission function. The error of the calculation was equal to 0.68% and 1.79% for graphene and graphane, respectively. A number of competitive advantages of the proposed approach compared to other methods in the literature are: Our approach does not use the Dijkstra method used in [9]. There is no need to construct a distance matrix and to find the shortest path in the plot. Also, the program implementation of our approach is simpler. Besides, our approach ensures a higher acceleration rate in calculations of the transmission functions of polyatomic structures. In particular, by the example of the graphene fragment, it was shown that the calculation speed of transmission function using our approach is three times higher than the calculation speed in the method proposed in [9]. Also, we introduce an additional part in the runtime of calculations, not only at the post-processing stage. The limitations of our method are that a too coarse *k*-point sampling or a too coarse energy sampling will lead unrealistic results, in spite of any post-processing scheme.

Using developed method we obtained new knowledge about the electrical conductive properties of a new composite material, namely pillared graphene. The calculated electrical conductance and resistance of the pillared graphene film showed that the current flow is more preferable along the zigzag edge of the graphene sheet both for single-layer and two-layer composites. The average resistance value in this direction was 12 kΩ for the single-layer composite. This value is close to the resistance value of an ideal nanotube. For the two-layer composite, the average resistance was 10.2–29.3 kΩ depending on the direction. This can be explained by the high electrical conductance of graphene nanostructures with a zigzag edge. The conductance of these ribbons does not depend on the width of the ribbon and weakly depends on its topology. In the direction of the armchair edge of the graphene sheet, the electrical conductance depends significantly on the topology of the film. The resistance changes drastically with the increase in CNT length. Using the analogy with graphene nanoribbons, it can be seen that the regularities in the electronic transport along the armchair edge are determined by the width of the ribbon and its morphology. In summary, we can conclude that the pillared graphene films with nanotubes having a diameter of 1.23 nm are characterized by a relatively high electrical conductivity. Due to high strength and conductivity, these films, provided a developed surface and pores for filling with the necessary connections, could be successfully applied in electronic devices and as electrodes of storage batteries.
